# Characterization of three-dimensional field distribution of bowtie aperture using quasi-spherical waves and surface plasmon polaritons

**DOI:** 10.1038/srep45352

**Published:** 2017-03-30

**Authors:** Changhoon Park, Howon Jung, Jae W. Hahn

**Affiliations:** 1 Nano Photonics Laboratory, School of Mechanical Engineering, Yonsei University, 50 Yonsei-ro, Seodaemun-gu, Seoul 120-749, Republic of Korea

## Abstract

We present an analytical formula to predict the three-dimensional field distribution of a nanoscale bowtie aperture using quasi-spherical waves (QSWs) and surface plasmon polaritons, which are excited by the fundamental waveguide mode and local plasmons of the aperture, respectively. Assuming two separate bowtie apertures in a metal film, we analysed the decay characteristics of QSWs using a finite difference time-domain method. To verify the formula, we recorded the spot patterns of the bowtie aperture on a photoresist film using various exposure times, and fit the patterns to the analytical formula in terms of the width and depth of the patterns. In addition, it was found that the formula successfully represented the dipole characteristics of the spot patterns, which were in agreement with the surface geometry, with a root-mean-square error of 9.4%. We expect that our theoretical formula will extend the potential applications of nanoscale bowtie apertures to plasmonic device fabrication, three-dimensional plasmonic lithography, and other technologies.

With the observation of extraordinary transmission (EOT) in small-hole arrays, the underlying physics of the optical response of a metallic hole at optical and near-infrared frequencies has attracted interest^1^,^2^. Surface plasmon polaritons (SPPs) are considered one of the main factors for high transmission, and experiments have been conducted to verify that they are the main source of high transmission^3^,^4^. However, in a single-hole or slit aperture, because SPPs are excited by diffraction at the edge of the aperture, the SPP excitation efficiency largely depends on the geometry of the aperture, and could vary^5^. To explain the difference between experimental results and the SPP model, a diffracted field without SPPs has been introduced as an extra contribution to high transmission. It is called a quasi-cylindrical wave (QCW) in a slit aperture^6^. QCWs are excited by a line dipole source, and the attenuation rate of QCWs on a metallic surface is different from that of general cylindrical waves in a real metal^7^. QCW characteristics are well reported in the literature^8^,^9^,^10^.

Because of its strong field confinement and anomalously high transmission, the ridge aperture has been used for nano-lithography^11^,^12^, optical tweezers^13^,^14^, and data storage^15^. Surface charges accumulate near the edges of a ridge aperture and act as a point dipole source. These are called local plasmons^16^. In addition, the longer wavelength of the ridge aperture allows a waveguide mode and facilitates a larger transmission compared with a circular aperture^17^. More specifically, a ridge aperture consists of an open arm and sharp ridge. The open arm and surface plasmon generated at the ridge wall induces a high effective index inside the aperture, and the cutoff wavelength becomes large. Thus, the far-field or propagating field can pass through the aperture^17^. For practical applications, many researchers have focused on the spectral resonance of the ridge aperture in terms of its geometrical dimensions^18^,^19^. From another point of view, extending a ridge aperture, such as through three-dimensional surface patterning, requires an analytical description of the field distribution around it. In spite of its significance, the underlying physics of the field distribution by the ridge aperture has not been well investigated.

In this paper, we propose an analytical formula to predict the three-dimensional field intensity distribution around a bowtie aperture, which is one type of ridge aperture. In order to describe the surface waves by the bowtie aperture, we extended the QCW concept to quasi-spherical waves (QSWs) in a two-dimensional hole-type aperture, such as a circular hole or ridge aperture. In order to simplify the problem, we used the empirical solution to predict the QSW attenuation rate. We expected local plasmons to excite Hankel-type SPPs and QSWs, and for them to have a cosine dependence on the azimuthal angle. In a surface wave analysis using the finite difference time-domain (FDTD) method, we determined that the QSWs had an asymptotic attenuation rate affected by the distance from the aperture in a real metal. We quantitatively verified that our formula fit the surface geometry of spot patterns recorded on a photoresist (PR) thin film.

## Results and Discussion

### Intensity distribution by ridge aperture

**Figure 1:**
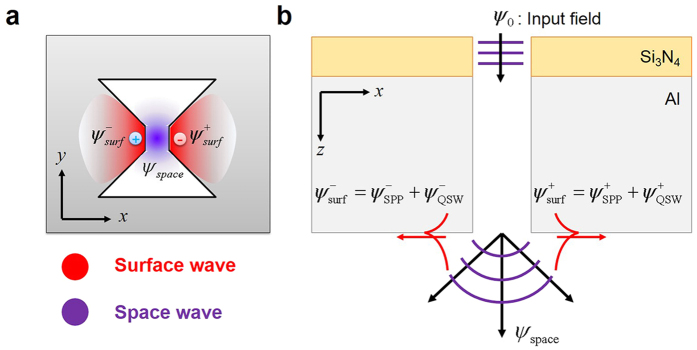
Schematic of the electromagnetic field in a ridge aperture in (**a**) the *xy*-plane and (**b**) the *xz*-plane. In Fig. 1, ψ represents the electromagnetic field vector defined by [*E*_z_, *H*_y_]. The surface wave propagates on the metallic surface with cosine dependence, and the space wave propagates spherically because of the small ridge gap. The purple wave represents a field whose wavelength is equal to the incident wavelength; the red wave represents a field whose wavelength is shorter than the incident wavelength.

When light is incident on a ridge aperture, as shown in Fig. 1, two emission sources are generated underneath the aperture: one is a horizontal dipole source, which is local plasmon located on the metal-dielectric interface, and the other is a point source created by the field transmitted through the aperture. The horizontal dipole excites surface waves composed of the evanescent mode of QSWs and SPPs.

For a perfect conductor, the evanescent mode of the QSW has a damping rate of *ρ*^−1^, which is the same as the attenuation rate of a spherical wave. For a real metal, however, the decay rate of QSW is similar to the decay rate of a spherical wave in the vicinity of the aperture, and this wave has an asymptotic *ρ*^−2^ attenuation over a long distance from the aperture^7^,^20^. In the intermediate regime, we expect the evanescent mode of the QSW to fall off with increasing distance (as *ρ*^−*q*^). Thus, with *φ* = 0, the magnetic field on the surface, *H*_*y*_ = *H*_SPP_ + *H*_QSW_, can be written as


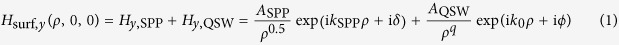


where *δ* is an arbitrary phase constant of the SPP, *ϕ* is an arbitrary phase constant of the QSW, *A*_SPP_ is the SPP magnetic field amplitude, *A*_QSW_ is the QSW magnetic field amplitude, and *ρ, φ, z* are the cylindrical coordinates. With Maxwell’s curl equation applied to the evanescent mode of the QSW, the electric field on the surface is represented by


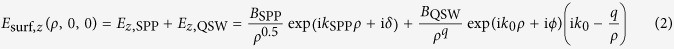


where *B*_*S*PP_ is the electric field amplitude of the SPP, and *B*_QSW_ is the electric field amplitude of the QSW. We assumed that the electric field and magnetic field have a harmonic dependence on time, i.e., in the form of exp(*−*i*wt*). As shown in Fig. 1, the electromagnetic field of the surface wave has a cosine dependence of *φ*, and it can be achieved by multiplying both equations (1) and (2) with cos *φ*.

With equations (1) and (2), the intensity of the surface wave can be expressed as


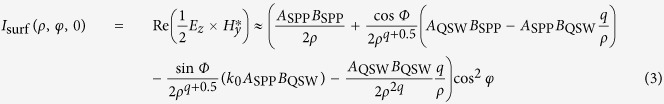


where *E*_*z*_ is the *z* component of the electric field; *H*_y_^*^ is the complex conjugate of the magnetic field on the surface, *H*_*y*_; *Φ* is the phase delay between the evanescent modes of the QSW and SPP; and *ρ, φ, z* are the cylindrical coordinates. In order to derive equation (3), we used *k*_SPP_ ≒ *k*_0_ and disregarded the difference between the wave vectors of the SPP and QSW. The cosine term in equation (3) originates from the dipole source.

In addition to the surface wave, the space wave defined as the propagating mode of the QSW and transmitted field also contributes to the field distribution. Assuming that the ridge gap is so small that the source of the space wave can be regarded as a point source, the irradiance from the fundamental mode of the waveguide follows the inverse square law:


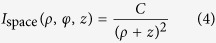


where *C* is a constant related to the space wave^18^.

### Evaluation of QSW decay rate

We calculated the QSW decay rate with FDTD (Lumerical 8.12.590) simulation. For experimentalists or some theoreticians in the field of nano-apertures, FDTD simulations are somewhat popularly used to analyse the electromagnetic field distribution of the bowtie aperture, slit aperture, and other waveguide because the FDTD solves Maxwell’s equations numerically^16^,^21^,^22^,^23^.

**Figure 2:**
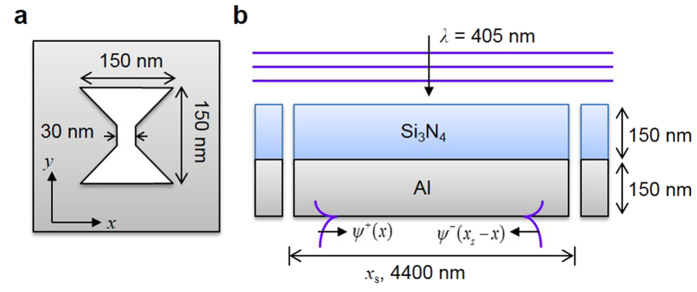
Simulation geometry for characterizing the decay rate of a QSW: (**a**) geometry of a single ridge aperture; (**b**) two apertures separated by distance of *x*_*s*_ in a dielectric/metal layered structure.

To simulate a situation in which both a QSW and SPP are generated underneath a bowtie-shaped aperture, we assumed a metal film with two bowtie apertures separated by a distance of *x*_s_. Figure 2 shows a schematic of the FDTD simulation geometry for extracting the QSW from the total field. In the FDTD analysis, we used an incident light wavelength of 405 nm, a metal layer of aluminium with a permittivity of −23.9819 + 4.9508i, and a dielectric layer of silicon nitride with permittivity of 4.2845; *x*_s_ was set at 4400 nm to meet the condition of a negligibly small QSW. We normalized the incident electric field by *E* = 1 and the magnetic field *H* = 1/*Z*_0_, where *Z*_0_ is the vacuum impedance, denoted by (*μ*_0_/*ε*_0_)^1/2^.

To estimate the QSW decay rate, we extracted the QSW from the total field. To separate the QSW from the surface wave, we used the interference of the surface wave by placing two ridge apertures separated by *x*_s_ of 4400 nm, which is larger than 10 *λ*. In the vicinity of the central point between the two ridge apertures, we expect interference of SPP waves propagating in opposite directions along the surface where the SPP is much larger than the QSW, because of the latter’s fast attenuation. The SPP field can then be obtained in the overall region. By subtracting the SPP from the total field, we can obtain the field distribution of the QSW in the FDTD simulation.

**Figure 3:**
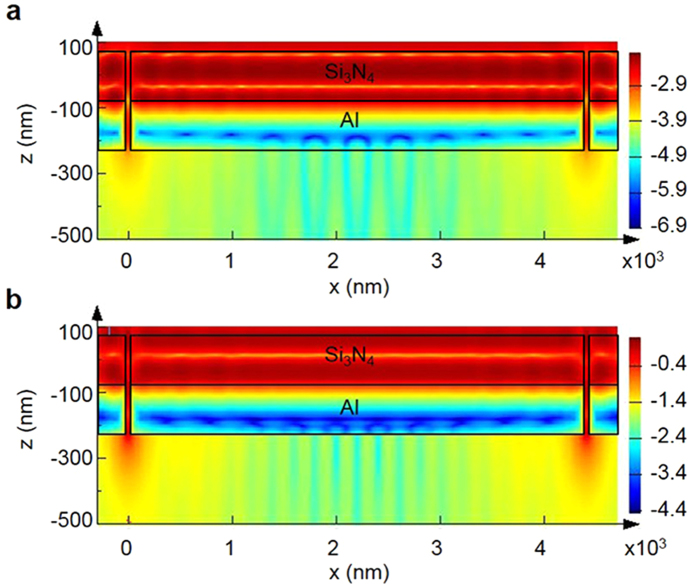
Calculated electromagnetic field diffracted by two ridge apertures. Ridge apertures are located at *x* = 0 and *x* = 4400 nm. (**a**) *y* component of the magnetic field in logarithmic scale. (**b**) *z* component of the electric field in logarithmic scale.

Figure 3 shows the results of the FDTD simulation for the geometry shown in Fig. 2. As can be seen in Fig. 3a, light illumination on a subwavelength metallic ridge aperture results in horizontal electric dipole radiation, which depends on the azimuthal angle on the surface. On the contrary, the *y* component of the magnetic field generated by the aperture is dominated by the fundamental mode of its waveguide, and it is independent of the azimuthal angle. In order to analyse the surface wave along the metal surface, we focused on the field distribution along the *x* axis (*y = *0) on the metal-air interface.

At the interface between the metal film and air, the magnetic field of the QSW falls off as a function of *x*^−*q*^, where *q* is a factor determining the decay rate of the field. In order to fit the FDTD simulation results with the fitting equation, we adopted the sinusoidal function instead of Euler’s form. The electromagnetic field along the *x* axis on the surface can be written as


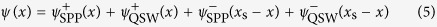


where *ψ* is the electromagnetic field vector defined by [*E*_z_, *H*_y_], *ψ*_SPP_ is the electromagnetic field for the SPP, *ψ*_QSW_ is the electromagnetic field for QSW, and the sign is + and − for increasing and decreasing, respectively, in the *x* direction. Far from the ridge aperture, the electromagnetic field is dominated by the SPP because of the fast attenuation of the QSW. We speculate that the QSW is much smaller than the SPP and can be disregarded at a distance above 1.6 μm. For 1.6 μm < *x* < 2.8 μm, the electromagnetic field can be as approximated by


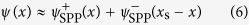


**Figure 4:**
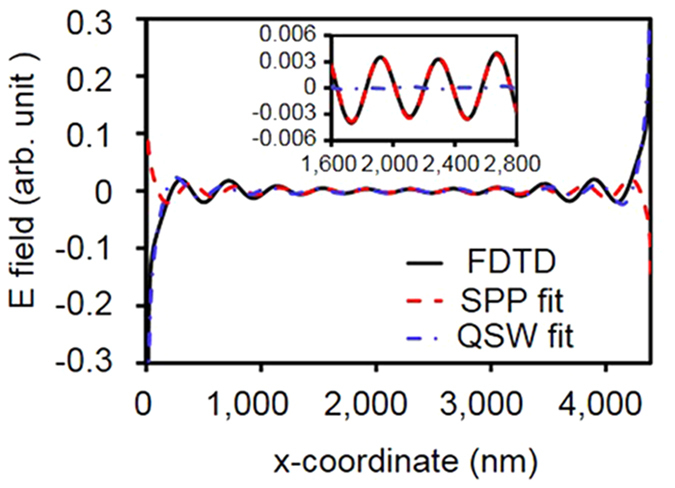
Electric field distribution obtained by FDTD simulation and fitting curve. The inset shows the electric field distribution in the region where the SPP is much larger than the QSW.

In order to predict the SPP field distribution, we used the FDTD simulation and fitted the numerical results with equation (6). The electric field of the SPP waves in the region of 1.6 μm < *x* < 2.8 μm can be described by


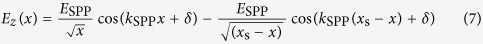


where *δ* is an arbitrary phase constant, and *E*_SPP_ is the amplitude of the SPP electric field. Owing to the dipole characteristics, the fields from the apertures at *x* = 0 and *x* = 4400 nm have different signs. Figure 4 shows the electric field distribution of the QSW and SPP. It is found that the FDTD simulation data is well matched with the fitted curve with equation (7) as shown in inset of Fig. 4. From the fitting process, we obtained *E*_SPP_ as 0.3544 and *δ* as −0.1209. To obtain the decay rate of the QSW, we subtracted the SPP contribution from FDTD simulation results in the entire region. From equations (2) and (5), the QSW can be described by


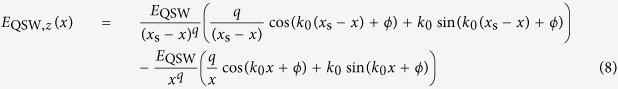


where *δ − ϕ* is a possible phase delay between the SPP and QSW, *q* is the decay rate, *E*_QSW_ is the QSW electric field amplitude, and *k*_0_ is the wave vector in free space. By fitting the subtracted data with equation (8), we obtained *E*_QSW_ = 1.07 × 10^−6^, *q* *= *1.18, and *ϕ = *0.44 from the fitted curve. From the fitting results, we speculate that the QSW falls off as a function of *x*^–1.18^ in the region where *x* < 11 *λ*.

### Spot pattern on the photoresist by the bowtie aperture

For the purpose of visualizing the optical response of the bowtie aperture, we fabricated the spot pattern on the PR by using a plasmonic lithography system with the bowtie aperture^24^. Patterning on the PR depends on the exposure dose and threshold dose of the PR. The local exposure dose can be determined from the equation *D*_ex = _*It*, where *I* is the total intensity of the field, and *t* is the exposure time. The profile of the spot pattern matches the distribution where *D*_ex_ is equal to the threshold dose of the PR, *D*_th_. Thus, the field intensity distribution by the ridge aperture can be obtained by varying the input exposure time or intensity and measuring the pattern profile on the PR. To analyse the dose distribution resulting from the ridge aperture, we obtained the dose distribution resulting from both the surface wave and space wave. The sum of the two dose distributions is then the total dose distribution by the ridge aperture. To obtain the three-dimensional dose distribution, decay characteristics in the *z* direction are also required. From the definition of the surface wave, it consists only of the evanescent mode, representing the exponential decay in the *z* direction. On the contrary, the space wave with the real value of the free-space wave vector falls off as a function of 1/*z*^2^ in the *z* direction.

Assuming that the intensity distribution follows a Gaussian distribution, the exposure dose distribution by the surface wave on the PR can be described by


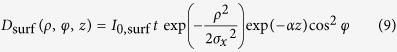


where *α* is the attenuation coefficient, *σ*_*x*_ is 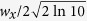, *w*_*x*_ is the full width at tenth of maximum (FWTM) in the *x* direction, and *I*_0,surf_ is the peak intensity due to the surface wave. For simplicity, we used the fact that *α* is the effective attenuation coefficient related with the SPP attenuation coefficient and the QSW evanescent mode. The dose distribution by the surface wave has a cosine dependence of the azimuthal angle because of the dipole radiation. Similarly, the exposure dose distribution by the propagating wave on the PR can be described by


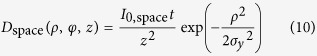


where *σ*_*y*_ is 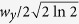, *I*_0,space_ is the peak intensity due to the space wave, and *w*_y_ is the full width at half maximum (FWHM) in the *y* direction.

In order to demonstrate the decay rate of the space wave and surface wave obtained from the FDTD method, we used the calibration curve, such as *w*_*x*_ and *w*_*y*_ in equations (9) and (10). The calibration curve is defined as the geometrical dimension of the spot pattern with respect to exposure dose. By imposing the threshold dose of the PR, the theoretical calibration curves for the *x*-width (*w*_*x*_) and *y*-width (*w*_*y*_) can be obtained. Compared with the *y*-width, which can be described by *w*_*y*_ = *f*(*D*_ex_), the *x*-width is mathematically complex. Thus, by taking the inverse function *D*_*ex*_ = *g*(*w*_*x*_), we can obtain the calibration curve for the *x*-width. To calibrate the system, we used the method reported in ref. 24. By varying the exposure time in increments of 1 ms and using a fixed light intensity, we obtained a spot pattern array. By measuring the geometrical features of each spot pattern with an atomic force microscope (AFM), we obtained the calibration data for the *x*-width and *y*-width.

By using the decay rate of intensity with the threshold dose condition, the depth, *x*-width, and *y*-width can be obtained. In this study, the *x*-width is defined as the FWTM of the intensity distribution in the *x* direction, and the *y*-width is defined as the FWHM of the intensity distribution in the *y* direction. Because the *x*-width is determined near the metal surface, the *x*-width is mainly determined by *E*_*z*_, which is related to the surface wave due to the boundary condition of the conductor. On the contrary, because the surface wave excited by the horizontal electric dipole does not affect the field intensity in the *y* direction, the *y*-width is determined only by the space wave.

**Figure 5:**
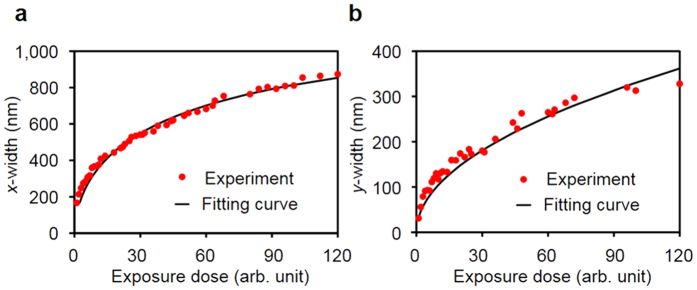
Calibration data for spot width. Experimental data (red circles) and fitting results (black solid line) are shown. (**a**) Calibration curve for the *x*-width, *w*_*x*_. (**b**) Calibration curve for the *y*-width, *w*_*y*_.

With the threshold dose and decaying rate of the spherical wave, the *y*-width can be calculated as


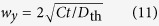


where *w*_*y*_ is the *y*-width, which is the FWHM in the *y* direction; *D*_*th*_ is the threshold dose on the photoresist. By fitting the calibration data with equation (11), we obtained *C*/*D*_th_ = 272.75 m^2^/s. In addition, an implicit function of the *x*-width can be obtained from the decay rate of the surface wave intensity (from equation (3)) and threshold condition as follows:


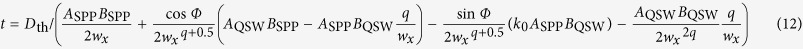


where *A*_SPP_ is the SPP magnetic field amplitude, *B*_SPP_ is the SPP electric field amplitude, *A*_QSW_ is the QSW magnetic field amplitude, *B*_QSW_ is the QSW electric field amplitude, *Φ* is the phase difference between the SPP and QSW, and *w*_*x*_ is the *x*-width, which is the FWTM in the *x* direction. Before we fitted the results with equation (12), we used *q* = 1.18 and *Φ* = −0.56, which were obtained from the FDTD simulation results with *D*_th_ fixed at a constant value of 1. From the fitting process with equation (12), we obtained *A*_SPP_ = 4.3, *B*_spp_ = −11.52, *A*_QSW_ = −1.64, and *B*_QSW_ = 1.78 × 10^−4^. Figure 5 shows the calibration data and fitting curve for the *x*-width and *y*-width.

**Figure 6:**
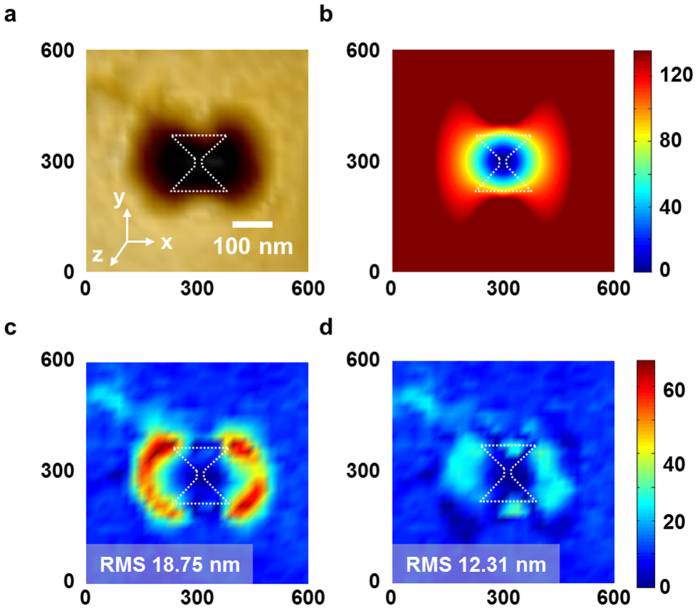
Simulation model and error distribution. (**a**) AFM image of the spot. (**b**) Simulated result of our analytical formula, which considers the dipole radiation effect at a spot depth of 130 nm for describing the experimental result shown in Fig. 3b. (**c**) Error distribution of the ellipsoidal Gaussian function. (**d**) Error distribution of our analytical formula.

With equations (9),(10),(11),(12), 13, 13, 14, we calculated the three-dimensional (3D) dose distribution by the bowtie aperture. By imposing the threshold dose condition on the total dose distribution, we obtained the three-dimensional spot shape in Fig. 6b. To assess the accuracy of the analytical formula, we used an ellipsoid described by an ellipsoidal Gaussian function as the reference model for comparison. Figure 6a shows an AFM image at a spot depth of 130 nm, and Fig. 6b shows the simulated result of our analytical formula, which considers the dipole radiation effects. Our analytical formula is close to reality, even though the effect of dipole radiation from the aperture creates an atypical shape of the spot. We defined the fitting error with the difference between the spot pattern and either the ellipsoidal Gaussian function or our analytical formula fitted to the spot pattern. Figure 6c and d show the error distribution of the ellipsoidal Gaussian function and our analytical formula, respectively. The analytical formula has a peak-to-valley (P-V) error value of 30.60 nm and a root-mean-square (RMS) error of 12.31 nm; the reference model has a P-V error of 67.65 nm and an RMS error of 18.75 nm. This means that the P-V and RMS errors in the analytical formula are 55% and 34% lower, respectively, than those of the reference model.

In summary, we presented an analytical formula of the 3D field intensity distribution by a bowtie aperture using the concept of QSWs and SPPs, which were generated by the fundamental waveguide mode and local surface plasmon polaritons of the aperture, respectively. Assuming two separate bowtie apertures on a metal film, we calculated the attenuation rate of the QSWs on the metallic surface using the FDTD method and obtained an asymptotic decay rate *q* of 1.18 for distance *x* < 11 *λ*. Using the spot patterns recorded on a photoresist film, we calibrated the formula in terms of the width and depth of the patterns and determined the coefficients for the analytical formula. Our analytical formula successfully reflected the dipole characteristics of the optical spots and was in close agreement with the surface geometry of the spot patterns, with an RMS error of 9.4%. We expect that our theoretical formula will extend the potential applications of nanoscale bowtie apertures to plasmonic device fabrication, three-dimensional plasmonic lithography, and other technologies.

## Methods

### Experimental system

The experimental setup of the plasmonic lithography system was shown in our previous work^24^. A continuous-wave diode laser with a wavelength of 405 nm was used as the light illumination source, and an objective lens (CFI LU Plan Epi El WE, Nikon; 100×, Na: 0.8) was used to focus the laser beam onto the aperture. The bowtie aperture was perforated in a silicon nitride layer with a thickness of 150 nm coated with a 150 nm aluminium layer. This configuration is well described in Fig. 2 for the case of one bowtie aperture.

## Additional Information

**How to cite this article:** Park, C. *et al*. Characterization of three-dimensional field distribution of bowtie aperture using quasi-spherical waves and surface plasmon polaritons. *Sci. Rep.* **7**, 45352; doi: 10.1038/srep45352 (2017).

**Publisher's note:** Springer Nature remains neutral with regard to jurisdictional claims in published maps and institutional affiliations.
